# Health promoting behaviour of medical versus non-medical students during COVID-19 pandemic: results from the COLA cross-sectional study

**DOI:** 10.1186/s12967-021-02899-y

**Published:** 2021-06-04

**Authors:** Julius Steffen, Jenny Schlichtiger, Stefan Brunner, Bruno C. Huber

**Affiliations:** Department of Medicine I, LMU-Klinikum, Ludwig-Maximilians-Universität München, Munich, Germany

**Keywords:** Physical activity, Smoking, COVID-19, Medical students, Health apps and wearables

## Abstract

To investigate the COVID-19 pandemic related alteration of health promoting behaviour during lockdown among medical students compared to other students.

In this cross-sectional study, we enrolled 1940 Bavarian students. Participants were asked to complete an online questionnaire 3 weeks after lockdown implementation, evaluating their lifestyle behaviour focusing on self-reported and objectively assessed physical activity.

1154 medical (59.5%) and 786 non-medical (40.5%) students were included (median age 22.0 [IQR, 20.0–25.0], 71.5% female). Physical activity decreased in both groups after lockdown implementation. During lockdown, medical students reported higher physical activity levels compared to non-medical students. This was corroborated by daily step count data assessed by wearables (median steps per day [IQR], 6979 [5218–9348] versus 6581 [4497–8491], p = 0.02). Smoking behaviour during lockdown did not differ between medical and non-medical students (increased in 11.8% vs 13.6%, decreased in 31.9% versus 36.9%).

During the COVID-19 pandemic, alteration of lifestyle behaviour among medical students was significantly different compared to non-medical students. This result suggests that medical students are more concerned about health promoting behaviour even in crisis situations.

## Introduction

Lockdowns implemented by governments all over the world during the current COVID-19 pandemic highly affected most people’s daily life. In Bavaria, the COVID-19 crisis hit during semester break. Bavaria was affected more severely by COVID-19 than other areas in Germany. Lockdown was implemented by the local government on the March 21, 2020 and included mandatory homestay, shutting gyms, and prohibiting other types of sports.

Physical activity (PA) is regarded crucial for physical and mental health in young and matured adults. A reduction of PA in the general population has been suspected during the COVID-19 crisis and could result in an increased cardiovascular risk [1]. High PA levels have been found to correlate with academic achievements among medical students [2] and to reduce stress levels [3]. Smoking habits vary widely between genders and countries [4] but are usually lower among medical students. Interestingly, physicians and medical students have been shown to be more concerned about health promoting behaviour than other students [5, 6]. During lockdown, in contrast to most other young adults, medical students were encouraged to continue internships and rotations. Further, they were often asked to volunteer for different positions at their teaching hospitals [7].

The continuation of sports and other health promoting activities during lockdown time is warranted and could be especially beneficial for medical students engaged in COVID-19 patient care. However, the effect of crises and pandemic lockdowns on students’ lifestyle is not well explored. Therefore, we aimed to investigate the pandemic-related alteration of health promoting activities among medical students compared to other students using an online survey.

## Materials and methods

Data analysed here were collected in the cross-sectional COLA study (retrospectively registered at clinicaltrials.gov, NCT04361877), an online survey among young adults in Bavaria [8]. The study was approved by the ethics committee of the Ludwig-Maximilians-University (LMU) Munich, Germany (“Ethikkommission der Medizinischen Fakultät der LMU”), approval number 20-268 KB, and was performed in accordance with the Declaration of Helsinki. Participation was voluntary. Participants were informed and consented to all recorded data being used for study purposes. The questionnaire collected information on participant’s demographics and their PA behaviour before and during lockdown. Physical activity was regarded as sports in general. Physical activity at work or commuting to work were not regarded as such. Step count data from smartphones or wearables should be provided for three consecutive representative days, if available. The survey was distributed among young adults enrolled at Bavarian universities via email and was active from March 30th to April 11, 2020.

Shapiro test was applied for normality assessment. Differences between the two groups of students were compared using Mann–Whitney–Wilcoxon test for continuous data and Fisher’s Exact test for categorial data. To calculate differences in step counts, Wilcoxon signed-rank test was used for within-group comparisons, and Mann–Whitney–Wilcoxon test was used for between-group comparisons. A p-value < 0.05 was regarded statistically significant for all tests. All statistical analysis was performed with RStudio, version 1.2.5033 (RStudio Inc., Boston, MA, USA), graphs were designed with Adobe Illustrator version 24.0.3 (Adobe Inc., San Jose, CA, USA).

## Results

Thousand nine hundred forty young adults from six Bavarian universities completed the questionnaire (return rate 24%), with 1154 medical students (59.5%) and 786 non-medical students (40.5%). Characteristics of study participants are shown in Table 1. Median age differed significantly between groups with medical students being 1 year younger than the group of other students. Most participating students were studying at one of the two universities in Munich (Ludwig-Maximilians-Universität, LMU, or Technische Universität München, TUM).

**Table 1:** Characteristics of study participants

	Medical (N = 1154)	Other (N = 786)	Total (N = 1940)	p value
Female gender	805 (70.6%)	566 (72.8%)	1371 (71.5%)	0.280
Age (years)	22.0 [20.0–25.0]	23.0 [21.0–25.0]	22.0 [20.0–25.0]	**0.001**
Age group				0.742
17–25 years	893 (77.6%)	610 (77.7%)	1503 (77.6%)	
26–35 years	246 (21.4%)	164 (20.9%)	410 (21.2%)	
36–50 years	12 (1.0%)	11 (1.4%)	23 (1.2%)	
BMI (kg/m^2^)	21.6 [20.1–23.4]	21.6 [20.1–23.7]	21.6 [20.1–23.4]	0.668
Smoker	61 (5.3%)	58 (7.5%)	119 (6.2%)	0.066
University town				
Munich	1147 (99.4%)	527 (67.0%)	1674 (86.3%)	
Erlangen–Nuremberg	4 (0.3%)	96 (12.2%)	100 (5.2%)	
Bamberg^a^	0 (0.0%)	79 (10.1%)	79 (4.1%)	
Augsburg^a^	0 (0.0%)	50 (6.4%)	50 (2.6%)	
Others	3 (0.3%)	34 (4.3%)	37 (1.9%)	
Discipline				
Medicine	1154 (100.0%)	0 (0.0%)	1154 (59.5%)	
Natural sciences	0 (0.0%)	257 (32.7%)	257 (13.2%)	
Humanities	0 (0.0%)	181 (23.0%)	181 (9.3%)	
Pedagogics	0 (0.0%)	123 (15.6%)	123 (6.3%)	
Psychology	0 (0.0%)	92 (11.7%)	92 (4.7%)	
Economics	0 (0.0%)	72 (9.2%)	72 (3.7%)	
Law	0 (0.0%)	49 (6.2%)	49 (2.5%)	
Others	0 (0.0%)	12 (1.5%)	12 (0.6%)	

Data are given as number (percentage) or median [inter-quartile range]. A p-value of  < 0.05 was regarded statistically significant

^a^The universities in the towns of Bamberg and Augsburg do not have a medical faculty or only recently built one

Medical students underwent SARS-CoV-2 swabs more often (9.0%, n = 104 vs. 2.3%, n = 18, p < 0.001), test results did not differ [positive result: medical students 0.4% (n = 5) vs. others 0.3% (n = 2), p = 0.288]. During lockdown, changes to smoking behaviour were similar: 11.8% of medical students vs. 13.6% non-medical students increased and 31.9% and 36.9%, respectively, decreased smoking. Most reported unchanged smoking habits.

**Fig. 1:**
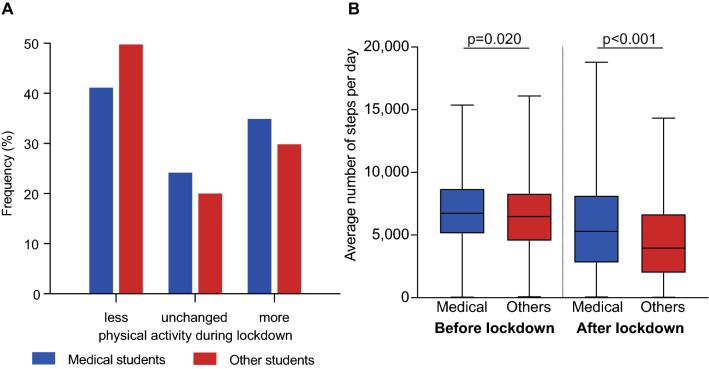
**A**, Perceived change in physical activity in medical students and non-medical students during lockdown. Participants were asked if the amount of physical activity they did since introduction of lockdown measures was more, less or unchanged compared to before. Medical students stated to have altered their physical activity slightly differently compared to non-medical students. 471 medical students (41.2%) decreased and 395 (34.5%) increased physical activity, compared to 390 non-medical students (49.9%) who decreased and only 233 (29.8%) who increased physical activity (p < 0.01). **B**, Daily step count during lockdown. Step count data from smartphones or wearables were provided for two representative time periods before, and during, lockdown. Since the introduction of lockdown, daily number of steps was significantly reduced in each group (p < 0.01). Already before lockdown, median daily step was significantly higher among medical students (median [IQR], 6979 [5218–9348] steps) compared to other students, (6581 [4497–8491] steps, p = 0.020). This difference was even more pronounced during the lockdown (medical students, 5469 [2865–8330] vs. other students, 4135 [2036–6858], p < 0.001)

Since the implementation of lockdown, 471 medical students (41.2%) decreased and 395 (34.5%) increased PA, compared to 390 other students (49.9%) who decreased and only 233 (29.8%) who increased PA (p < 0.001, Fig. 1A).

Weekly hours of PA, assessed on a 4-level scale (0 h, < 2 h, 2–5 h, and > 5 h), were similar between groups before lockdown. During lockdown, there was a significant difference (Table 2).

**Table 2:** Weekly hours of physical activity

	Medical students (N = 1154)	Other students (N = 786)	p value
Hours/week before lockdown			0.441
0 h	42 (3.6%)	36 (4.6%)	
< 2 h	285 (24.7%)	209 (26.6%)	
2–5 h	596 (51.7%)	381 (48.5%)	
> 5 h	230 (19.9%)	160 (20.4%)	
Hours/week during lockdown			< 0.001
0 h	78 (6.8%)	70 (8.9%)	
< 2 h	290 (25.1%)	269 (34.3%)	
2–5 h	466 (40.4%)	302 (38.5%)	
> 5 h	320 (27.7%)	144 (18.3%)	

Participants were asked to quantify the number of hours of PA in a representative week before and during lockdown. Data are given as number (percentage). A p-value of  < 0.05 was regarded statistically significant

The fraction of participants doing no sports was equally low at baseline (medical students, n = 42, 3.6%, vs. others, n = 36, 4.6%) and almost doubled (medical students, n = 78, 6.8%, vs. others, n = 70, 8.9%). Among sporty participants (> 2 h/week), a decrease of sports was seen in only 41% (n = 335) of medical students but in 56% (n = 300) of other students. Participants reporting > 5 weekly hours of sports increased in medical students from 19.9% (n = 230) to 27.7% (n = 320) while there was no relevant change among other students (from 20.4%, n = 160, to 18.3%, n = 144, Table 3).

**Table 3:** Change of hours of physical activity

Medical students (n = 1154)					
Number of participants (percentage before lockdown)		During lockdown			
		0 h	< 2 h	2–5 h	> 5 h
Before lockdown	0 h	13 (31.0%)	6 (14.3%)	17 (40.5%)	6 (14.3%)
	< 2 h	39 (13.7%)	62 (21.8%)	143 (50.2%)	41 (14.4%)
	2–5 h	22 (3.7%)	197 (33.1%)	218 (36.6%)	159 (26.7%)
	> 5 h	4 (1.7%)	25 (10.9%)	87 (37.8%)	114 (49.6%)
Other students (n = 786)					
Number of participants (percentage before lockdown)		During lockdown			
		0 h	< 2 h	2–5 h	> 5 h
Before lockdown	0 h	12 (33.3%)	14 (38.9%)	7 (19.4%)	3 (8.3%)
	< 2 h	23 (11.0%)	54 (25.8%)	108 (51.7%)	24 (11.5%)
	2–5 h	30 (7.9%)	167 (43.9%)	123 (32.4%)	60 (15.8%)
	> 5 h	5 (3.1%)	34 (21.3%)	64 (40.0%)	57 (35.6%)

Participants were asked to quantify the number of hours of PA in a representative week before and during lockdown. Shaded fields indicate unchanged amounts of PA

In both groups, a significant reduction of steps per day was found during lockdown compared to before (p < 0.01). When comparing the two groups, the median daily step count was significantly higher among medical students (median [IQR], 6,979 [5218–9348] steps) compared to other students, (6581 [4497–8491] steps, p = 0.02, Fig. 1B). The difference was more pronounced after the lockdown (medical students, 5469 [2865–8330] vs. other students, 4135 [2036–6858] steps, p < 0.01). When analysing this in more detail, out of all the disciplines, medical students had the highest median step count after lockdown introduction (data not shown). Medical students named running as a PA more often than others before (52.1%, n = 601 vs. 42.6%, n = 335, p < 0.01), and during lockdown (57.0%, n = 658, vs. 46.1%, n = 362, p < 0.01).

Subgroup analyses of students from the largest participating town, Munich, showed comparable results.

## Discussion

In this large-scale online survey, we found that lockdown measures during the pandemic significantly affected PA levels in young adults but had no relevant effect on smoking behaviour. Most study participants reduced PA during lockdown. Remarkably, PA behaviour of medical students was affected less compared to other students.

Results from a comparable questionnaire on PA and personal risk behaviour during normal times comparing medical students to law students have last been published almost 40 years ago [5]. Similar to our current results, running was among the most popular activities and medical students were found to go running more frequently than other students.

Medical students were regarded more sporty than other students. In addition to the former results, we could now emphasize this finding with objective step count data from wearables and smartphone apps. Daily step counts can be easily and accurately assessed by accelerometers, which are integrated in wearables, as well as in health and fitness apps on smartphones [9]. They have been established as an overall measure of PA and an excellent prognostic marker for all-cause mortality [10].

Medical students appear to be more concerned about personal health than others (5, 6). They are aware of the importance of PA as a major part of long-term disease prevention, which could explain a smaller reduction in PA compared to other students and fewer smokers. The higher rate of SARS-CoV-2-swabs among medical students further supports this assumption but could also be due to contacts to infected patients at the hospital. It is pleasing to see that the crisis resulted in a reduction of smoking in one third of all participants.

This is the first study to assess the effect of a pandemic on health promoting behaviour in medical students. Only Bavarian students mostly from the same town have been included in the study, limiting the generalizability. Due to the study size, conclusions on other study disciplines cannot be drawn. Its cross-sectional design does not allow further follow-up, which would be warranted as impaired cardiovascular prevention behaviour warrants further evaluation of long-term effects. Fortunately, in a cohort of medical students, negative effects of lockdown measures on preventive behaviour were not very pronounced.

## Data Availability

The datasets used and/or analysed during the current study are available from the corresponding author on reasonable request.

## Abbreviation

PA: Physical activity
